# Acute erythrodermic psoriasis complicated by systemic capillary leak syndrome following secukinumab therapy: a case report

**DOI:** 10.3389/fimmu.2026.1813490

**Published:** 2026-04-20

**Authors:** Ruisi Xu, Xinghua Gao, Li Zhang

**Affiliations:** 1 Department of Dermatology, The First Hospital of China Medical University, Shenyang, China; 2 Key Laboratory of Immunodermatology, Ministry of Education and NHC; National Joint Engineering Research Center for Theranostics of Immunological Skin Diseases, Shenyang, China

**Keywords:** apremilast, case report, cytokine storm, erythrodermic psoriasis, secukinumab, systemic capillary leak syndrome

## Abstract

A 66-year-old woman with a 6-month history of new-onset psoriasis after her first dose of COVID-19 vaccination recently progressed to acute erythroderma accompanied by fever and systemic inflammation. She was started on secukinumab for erythrodermic psoriasis (EP) after conventional therapies were contraindicated. On hospital day 6, her condition deteriorated with dyspnea, high fever, and edema. Laboratory evaluation revealed severe hypoalbuminemia and elevated inflammatory cytokines, with no evidence of infection. Imaging studies showed pulmonary edema and massive pleural effusions requiring therapeutic thoracentesis. After excluding sepsis, cardiogenic pulmonary edema, and allergic reactions, a diagnosis of systemic capillary leak syndrome (SCLS) complicating EP was established. Secukinumab was discontinued, and the patient received supportive care while being switched to apremilast. Her condition improved rapidly, with resolution of fever, reduction in pleural effusions, and significant diminishment of skin erythema. This case highlights a rare but life-threatening complication of severe acute EP, as both conditions are driven by a systemic cytokine storm. A single loading dose of secukinumab failed to effectively control the systemic cytokine storm in this patient, and the potential association between secukinumab administration and disease progression requires further investigation. We review the pathogenesis of EP, differentiate it from plaque psoriasis, identify the risk factors for SCLS, and discuss therapeutic considerations. Clinicians managing patients with severe psoriasis should remain vigilant for the potential occurrence of SCLS, particularly in those presenting with acute EP. Additionally, careful consideration should be given to the selection of biologic agents in high-risk patients.

## 1 Introduction

Erythrodermic psoriasis (EP) is a rare yet severe variant of psoriasis, accounting for less than 3% of cases (1). It is characterized by generalized erythema and scaling over more than 90% of the body surface area (BSA), often accompanied by systemic symptoms such as fever, lymphadenopathy, malaise, edema, and electrolyte disturbances (1). Unlike chronic plaque psoriasis, EP can present acutely and poses a potential life-threatening risk if not treated promptly (1). Acute EP flares may result in high-output cardiac failure, thermoregulatory disturbances, and a phenomenon known as a “psoriatic cytokine storm,” which refers to the overwhelming release of proinflammatory cytokines (2). These cytokine surges can lead to systemic complications resembling sepsis, including fever, capillary leakage, and multi-organ dysfunction. One of the most dangerous complications associated with EP is systemic capillary leak syndrome (SCLS). SCLS is an exceedingly rare disorder characterized by increased capillary permeability to proteins, causing protein-rich fluid to shift from the intravascular to the interstitial space (3). SCLS episodes can be classified as idiopathic, commonly known as Clarkson’s disease, or secondary SCLS, which may be triggered by various factors, including infections, drugs, toxic agents, hematological malignancies, and autoimmune diseases affecting either systemic or cutaneous systems (4). SCLS has been reported as a potentially fatal complication in patients experiencing acute flares of severe pustular or erythrodermic psoriasis (5–8). The management of EP poses significant challenges due to its rarity and acute clinical presentation. Traditional first-line systemic therapies, including cyclosporine, methotrexate, and retinoids, can be effective. However, each treatment carries inherent risks and may be contraindicated in patients with hypertension or hepatic dysfunction (9). Biologic therapies targeting TNF-α, IL-12/23, or IL-17 have demonstrated favorable outcomes in plaque psoriasis and are utilized off-label for EP (1). A recent meta-analysis has shown that IL-17-targeted biologics induce a more rapid and pronounced clinical response in the management of EP compared with other biologic agents (10). Secukinumab, a monoclonal antibody against IL-17A, has been reported to induce rapid skin improvement and has been documented in multiple case reports and case series as effective for EP (1, 10). However, the intense immune dysregulation associated with EP has raised concerns regarding atypical reactions. We present a case of fulminant EP in which secukinumab therapy was complicated by SCLS and discuss the implications for pathogenesis and clinical management.

## 2 Case presentation

A 66-year-old Chinese woman was hospitalized due to diffuse erythema and scaling of the skin, accompanied by high fever. Six months earlier, she developed new-onset psoriasis shortly after her first dose of COVID-19 vaccine. Initial treatments, including narrowband UVB phototherapy, oral Chinese herbal medicine, and various topical therapies, were ineffective in controlling her condition. Over the 10 days preceding admission, her psoriasis acutely progressed to exfoliative erythroderma, accompanied by severe pruritus and intermittent fever at home. Her medical history included type 2 diabetes mellitus diagnosed 5 years earlier and hypertension diagnosed 8 years earlier. She received regular insulin injections and oral nifedipine controlled-release tablets, with good glycemic and blood pressure control. There was no family history of psoriasis.

**Figure 1:**
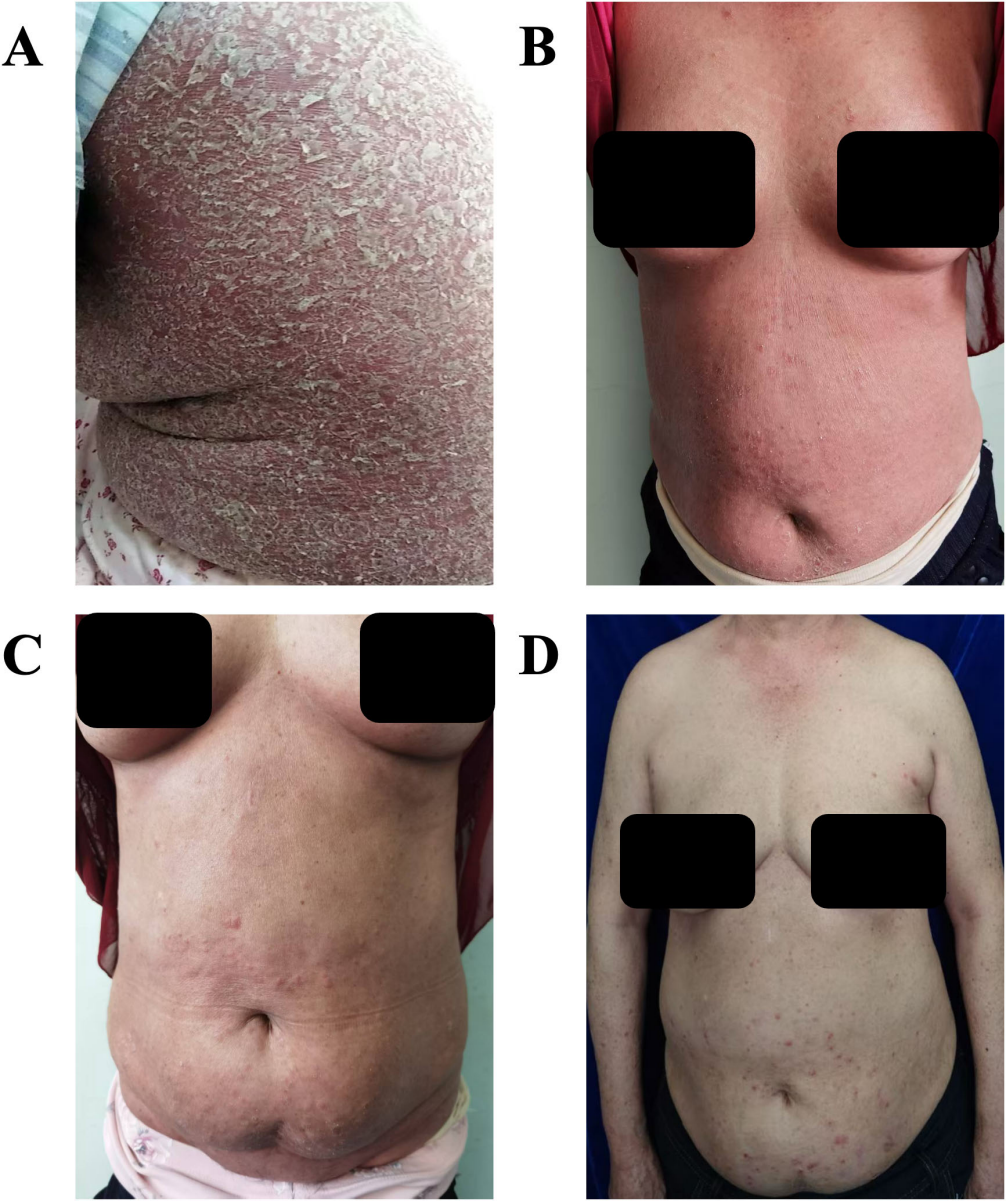
Clinical photographs. **(A)** on admission, **(B)** at 1-week outpatient follow-up, **(C)** at 4-week outpatient follow-up, **(D)** at 9-week outpatient follow-up.

**Figure 2:**
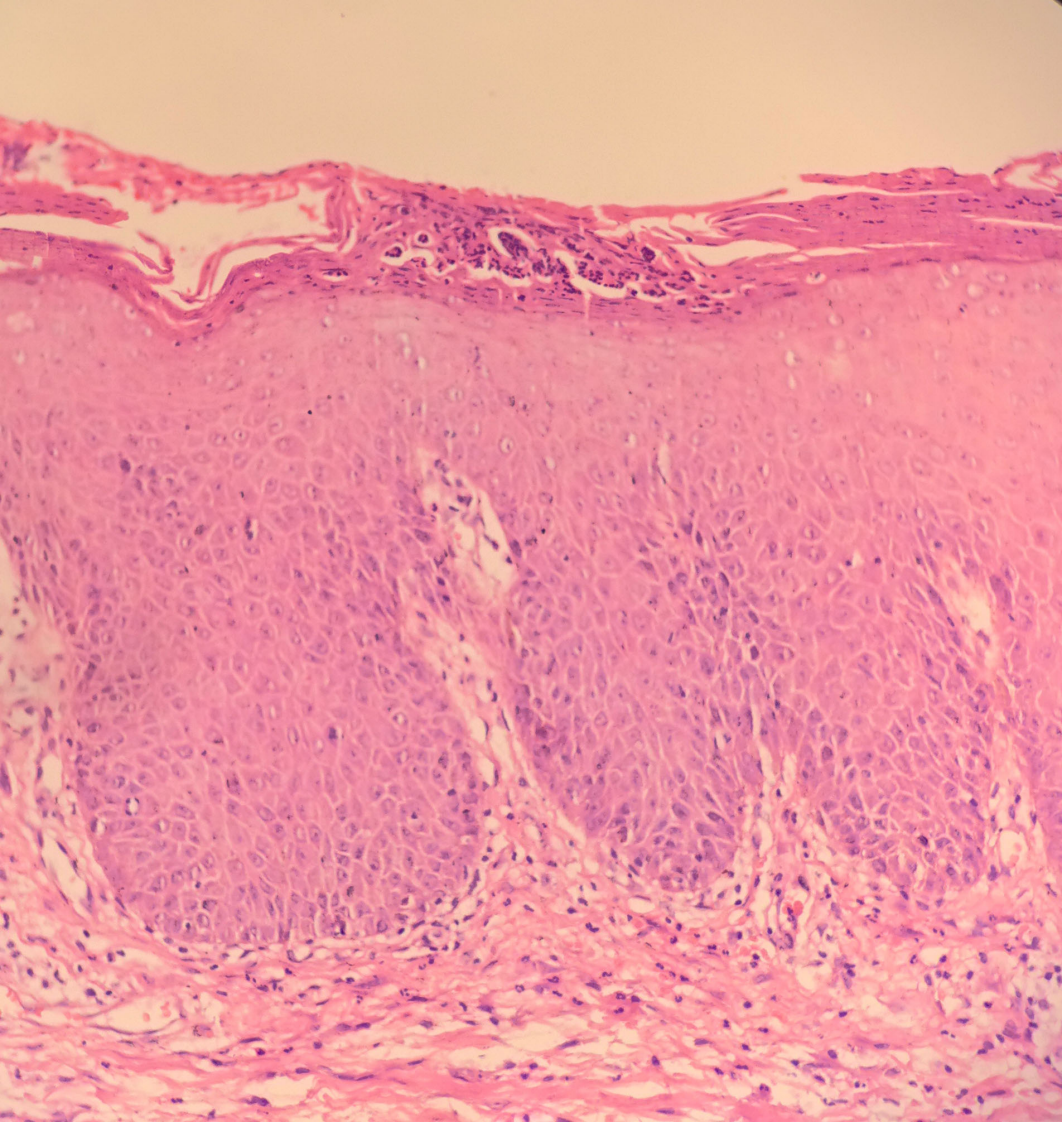
Histopathological examination showing neutrophilic microabscesses in the stratum corneum, psoriasiform epidermal hyperplasia, intercellular edema, vascular dilation, and perivascular lymphocytic and neutrophilic infiltrates in the dermis (hematoxylin and eosin, ×200).

Upon examination, the patient appeared ill, presenting with a temperature of 38.2 °C, a heart rate of 100 bpm, blood pressure of 150/75 mmHg, and a respiratory rate of 18 breaths per minute. She exhibited generalized erythema with desquamation affecting 100% of her BSA (**Figure 1A**). Mild pitting edema was observed in the lower extremities, along with tender axillary lymphadenopathy. Her Psoriasis Area and Severity Index (PASI) score was 49. Laboratory tests revealed significant inflammation, with C-reactive protein (CRP) at 81.67 mg/L (normal range: 0-8), interleukin-6 (IL-6) at 116.16 pg/mL (normal range: ≤20), IL-10 at 7.57 pg/mL (normal range: ≤5.9), and tumor necrosis factor-alpha (TNF-α) at 10.24 pg/mL (normal range: ≤5.5). D-dimer levels were elevated at 3.08 mg/L (normal range: <0.55). Liver enzymes were mildly elevated, with γ-glutamyl transferase (γ-GT) at 229 U/L (normal range: 7-45), alkaline phosphatase (ALP) at 160 U/L (normal range: 50-135), and aspartate aminotransferase (AST) at 45 U/L (normal range: 13-35). Notably, she presented with hypoalbuminemia at 29.9 g/L (normal range: 40–55), while renal function, complete blood count with eosinophil count and percentage, and procalcitonin levels remained within normal limits. No atypical lymphocytes were detected in the peripheral blood. Infectious workup upon admission returned negative results. Thoracic computed tomography (CT) and electrocardiogram findings were unremarkable, and abdominal ultrasound results were normal. A skin biopsy of the abdominal rash revealed psoriasiform epidermal hyperplasia with parakeratosis and neutrophilic microabscesses in the stratum corneum, intercellular edema, vascular dilation, and perivascular lymphocytic and neutrophilic infiltrates within the dermis (**Figure 2**). These histopathological features corroborated the clinical diagnosis of erythrodermic psoriasis. Given the acuity and severity of her EP, rapid-acting systemic treatment was necessary. Cyclosporine was avoided for hypertension and elevated liver enzymes, while methotrexate and acitretin were contraindicated due to hepatic dysfunction and potential toxicity. Secukinumab was thus selected for off-label use, with a 300 mg subcutaneous loading dose administered on hospital day 2. Concurrently, we provided supportive care, including oral glutathione for hepatic support, intravenous albumin infusions for hypoalbuminemia, and prophylactic antibiotics (levofloxacin) due to her fever and compromised skin barrier.

**Figure 3:**
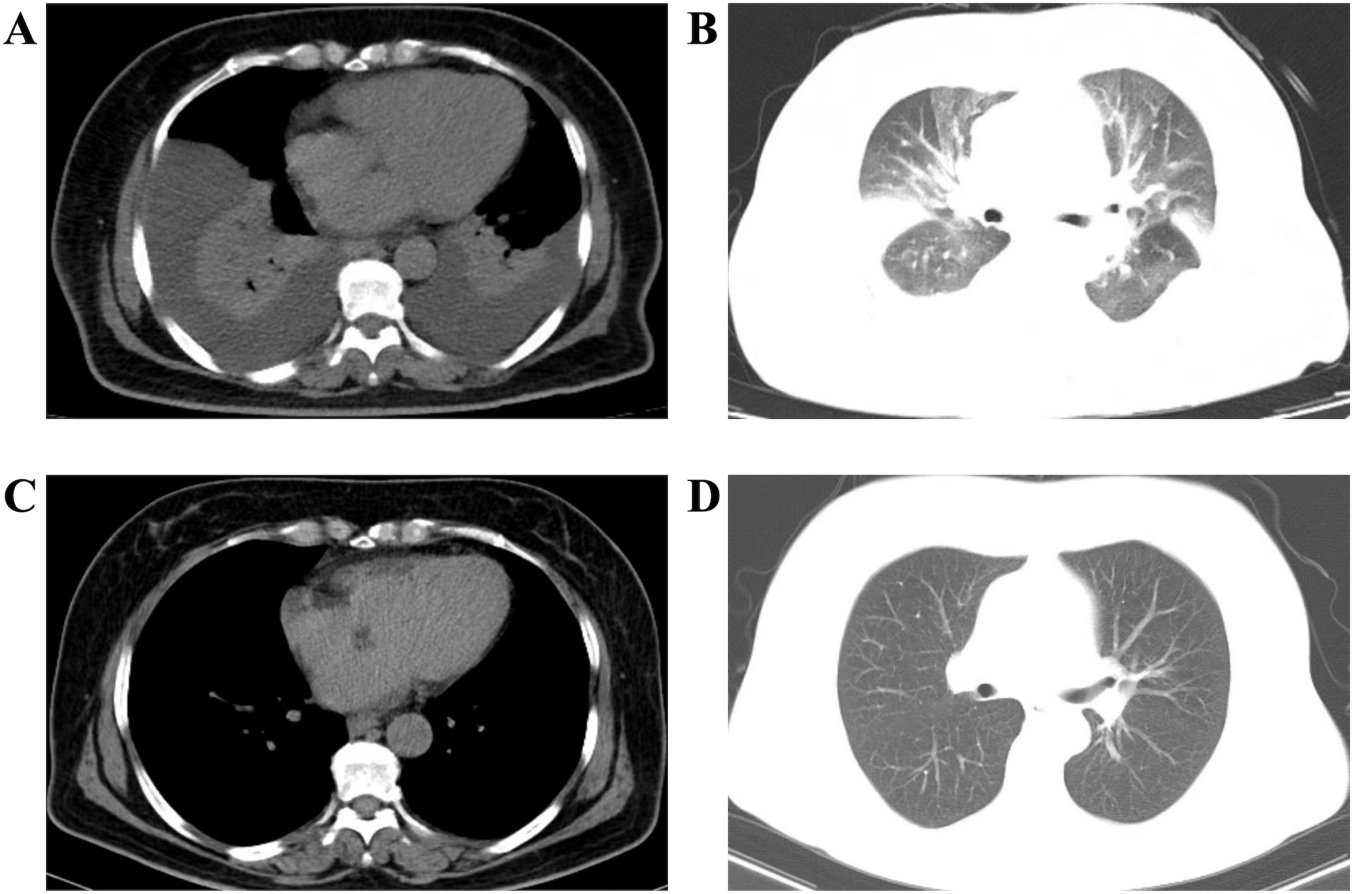
Thoracic CT showing massive bilateral pleural effusions and interstitial pulmonary edema [**(A)** mediastinal window, **(B)** lung window]. After treatment with apremilast, a repeat chest CT showed complete resolution of pleural effusions and pulmonary edema [**(C)** mediastinal window, **(D)** lung window].

On hospital day 6 (4 days after secukinumab administration), the patient’s skin erythema and scaling persisted. Her condition acutely worsened, accompanied by a fever of 39.2 °C, progressive dyspnea, and tachypnea. Examination revealed generalized pitting edema, which had worsened since admission, and new bilateral basal crackles on lung auscultation. Repeat laboratory tests indicated a further drop in serum albumin to 29.0 g/L. Liver function deteriorated (γ-GT 568 U/L, ALP 283 U/L, AST 48 U/L). Inflammatory markers remained elevated, with CRP at 159.79 mg/L and IL-6 at 257.67 pg/mL. However, the white blood cell, neutrophil, eosinophil, hematocrit, and hemoglobin levels were all normal, and procalcitonin levels remained low. Autoimmune serologies returned negative results. An urgent thoracic CT scan revealed massive bilateral pleural effusions accompanied by compressive atelectasis and interstitial edema in both lungs (**Figures 3A, B**). An arterial blood gas analysis conducted on room air indicated a PaO_2_ of 44 mmHg, signifying severe hypoxemia, with an SaO_2_ of 81%. Notably, cardiac evaluation yielded normal results. Echocardiography demonstrated preserved left ventricular function with no pericardial effusion, and cardiac enzymes as well as brain natriuretic peptide (BNP) levels were not elevated. These findings suggested a diagnosis of non-cardiogenic pulmonary edema. The leading consideration was a capillary leak process associated with her severe inflammatory state. Following consultations with the departments of Respiratory Medicine, Cardiology, Gastroenterology, Infectious Diseases, and Critical Care Medicine, the patient was transferred to the intensive care unit for closer monitoring. Broad-spectrum antibiotics, specifically cefoperazone-sulbactam, were started empirically due to the persistent high fever, pending repeat culture results. Aggressive supportive therapy was initiated, including supplemental oxygen, intravenous albumin, and diuretics (furosemide). A therapeutic thoracentesis was performed, with nearly 900 mL of straw-colored pleural fluid removed from the right hemithorax over 48 hours, leading to significant improvement in her breathing. Fluid analysis revealed transudative characteristics with no malignant cells identified. Cytological examination revealed a nucleated cell count of 170/uL. The differential counts showed neutrophils 47%, lymphocytes 23%, mesothelial cells 11%, eosinophils 0%, and macrophages 19%. Gram stain and cultures of the effusion were negative. No organisms were identified on acid-fast bacillus and fungal stains of the pleural fluid, and blood cultures showed no growth. These findings, along with normal procalcitonin levels, rendered infection unlikely.

On hospital day 8, the patient’s fever significantly decreased with a maximum temperature of 37.7 °C, and her oxygenation improved with SaO_2_ greater than 95% on 3 L of oxygen. After excluding sepsis, cardiogenic pulmonary edema, and allergic reactions, a diagnosis of SCLS in the setting of acute EP was established. We discontinued secukinumab due to concerns that it might have played a role in the development or exacerbation of SCLS. We initiated apremilast as an alternative systemic agent at 10 mg orally once daily, titrated to 30 mg twice daily over one week. The patient continued to receive supportive care, including topical emollients, supplemental oxygen, intravenous glutathione, intravenous albumin, and diuretics. The patient’s recovery was remarkable. By day 14, her respiratory status had stabilized. Repeat chest CT revealed a substantial reduction in pleural effusions, and CRP and IL-6 levels had decreased. She was discharged on apremilast 30 mg twice daily and oral glutathione.

**Figure 4:**
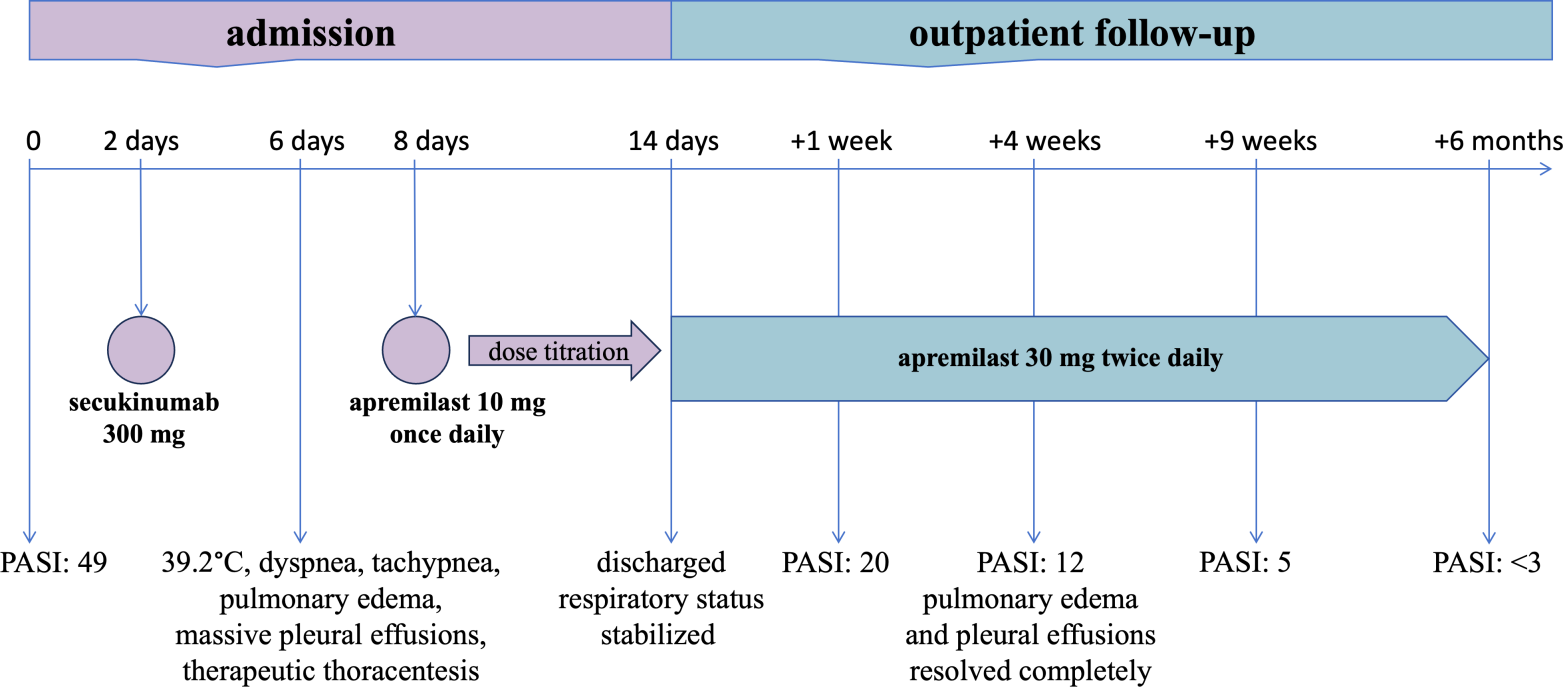
Timeline of administration for secukinumab and apremilast with relevant clinical manifestation during hospitalization and outpatient follow-up.

At one-week follow-up, the patient’s erythema and desquamation improved >50%, and her PASI score decreased to 20 (**Figure 1B**). At four-week follow-up, her liver enzymes normalized, her PASI score further decreased to 12 (**Figure 1C**), and she showed no residual pulmonary edema or pleural effusion (**Figures 3C, D**). By the nine-week follow-up, her erythroderma had almost resolved, and her PASI score had dropped to 5 (**Figure 1D**). She remained afebrile and in good condition. During six months of outpatient follow-up, there was no recurrence of erythroderma or capillary leak syndrome. Her psoriasis was controlled as mild plaque-type psoriasis with a PASI score of less than 3 while receiving apremilast (**Figure 4**).

## 3 Discussion

EP shows extreme immune dysregulation compared with classic plaque-type psoriasis. Plaque psoriasis is driven primarily by the Th1 and Th17 pathways, with high levels of TNF-α, IL-17, and IL-23 promoting keratinocyte proliferation (11, 12). By contrast, evidence suggests EP involves a shift towards a Th2-dominant cytokine profile (13). In EP, Th2 cytokines are expressed at a higher level, and the Th1/Th2 ratio is significantly lower than that in plaque psoriasis (13). The serum immunoglobulin E levels was found to be higher in EP than in psoriasis vulgaris (14). One analysis of EP patients demonstrated marked elevation of IL-4 and IL-13, as well as high levels of IL-6 and vascular endothelial growth factor (VEGF), indicating an aberrant immune response accompanied by upregulation of angiogenic factors (15). Vascular alterations play a critical role in the pathogenesis of psoriasis. A study has demonstrated that EP is characterized by increased vessel count, marked vascular dilation, and upregulated expression of intercellular adhesion molecule 1 (ICAM-1) (16). These observations reveal enhanced Th2 activity and significant dysregulation of angiogenic factors as hallmarks of EP, which can contribute to widespread vasodilation, edema, and the capillary leak phenomenon. Systemic inflammation, often accompanied by an exaggerated cytokine storm, is a defining feature of EP that differentiates this subtype from other subtypes of psoriasis (2, 17). Clinically, EP is often associated with systemic inflammatory response signs, whereas plaque psoriasis is typically more localized to skin with minimal systemic symptoms. EP can be further classified into two subtypes based on disease progression rate, namely slowly progressive and rapidly progressive. The latter refers to rapid-onset generalized erythema without distinct psoriatic plaques, and is associated with more severe systemic inflammatory responses, poor prognosis, and notable mortality (18, 19).

The real incidence of SCLS remains undefined, primarily because of its rarity and the common problem of delayed or mistaken diagnosis in clinical settings (4). The pathogenesis of SCLS remains obscure. Regardless of clinical context, hypercytokinemia is regarded as the underlying factor triggering capillary leak (3). Breakdown of endothelial adherens junctions (AJ) may be the key process by which cytokines augment vascular permeability (3). Elevated levels of endothelial factors like VEGF and angiopoietin-2 have been implicated in causing endothelial dysfunction and hyperpermeability (3). In severe psoriasis subtypes including erythrodermic and pustular psoriasis, massive inflammation is thought to act as the trigger for capillary leak events. Additionally, elevated levels of ICAM-1 are also involved in increasing microvascular permeability (20). Notably, EP and SCLS share a cytokine storm-driven systemic inflammation.

A range of drugs including IL-2, IL-11, IL-12 and monoclonal antibodies have been implicated in the induction of SCLS (3), as they can acutely activate large populations of immune cells and drive a rapid elevation of various cytokines, which is known as cytokine release syndrome (21). The median latency from initiation of the suspected therapy to SCLS onset is 8 days, with an interquartile range of 2.25-31.7 days (22). Secukinumab is an effective biologic agent for moderate-to-severe plaque psoriasis, and growing evidence supports its use in EP (1, 10, 23, 24). However, safety data in EP are limited. Secukinumab’s clinical trials did not report capillary leak syndrome, but rare serious adverse events have been reported in the post-marketing setting. A 2025 report described eosinophilic pleural effusion in a psoriasis patient treated with secukinumab, likely due to a hypersensitivity reaction (25). Both brodalumab and secukinumab, anti-IL-17 agents, have also been associated with eosinophilic pleural effusion in pustular psoriasis (26). Secukinumab may further induce erythema multiforme-like eruptions in generalized pustular psoriasis (27). These cases indicate that atypical inflammatory or hypersensitivity reactions can occur with IL-17 inhibitor therapy. However, the relationship between secukinumab and SCLS remains to be fully elucidated. Currently, there is no definitive clinical evidence for a direct causal link between secukinumab and the development of SCLS. The complication observed in this patient is hypothetical, and similar adverse events may potentially occur with other immunomodulatory or biologic therapies used for erythrodermic psoriasis.

Drug-induced hypersensitivity reaction may also present as SCLS (28). In our patient, to secukinumab, along with glutathione, levofloxacin, and albumin were all administered 4 days prior to the onset of SCLS. Based on the temporal association, all these agents were initially considered potential causative drugs. Glutathione and albumin were ruled out, as their continued use did not exacerbate SCLS, leaving secukinumab and levofloxacin as potential suspects. However, the absence of typical allergic skin lesions, lack of eosinophils in the pleural effusion, and persistently normal peripheral blood eosinophil counts on repeated testing excluded hypersensitivity reactions induced by these two drugs.

The clinical presentation of our patient, characterized by fever, lymphadenopathy, lower extremity edema, hypoalbuminemia, and markedly elevated CRP and IL-6 levels on admission, exemplified an acute fulminant EP that predisposed her to SCLS even before treatment initiation. Given that CRP and IL−6 levels increased significantly 4 days after secukinumab administration compared with admission levels. We consider that a single loading dose of secukinumab failed to effectively control the cytokine storm, leading to disease progression.

Idiopathic SCLS is defined by hypotension, hypoalbuminemia, and hemoconcentration (4). In contrast, secondary SCLS often lacks the complete diagnostic triad. Its main features include diffuse pitting edema, exudative serous cavity effusions, noncardiogenic pulmonary edema, acute respiratory distress syndrome (ARDS), acute kidney injury, hypotension, shock, and hypoalbuminemia. Hemoconcentration is frequently absent in secondary SCLS (3, 4). The absence of hemoconcentration in this case supports the diagnosis of SCLS secondary to EP. The pleural effusion in this patient exhibited transudative features, most likely secondary to the profound serum protein depletion.

SCLS currently lacks standardized diagnostic criteria and broadly validated biomarkers (4). It is a diagnosis of exclusion, relying on nonspecific symptoms and the exclusion of mimicking conditions, such as sepsis, angioedema, other drug reactions, and anaphylactic shock (3, 4). Distinguishing a psoriasis-related cytokine storm from true infection or sepsis is critical, and features such as marked elevation of CRP and IL-6 with normal procalcitonin and negative cultures, as seen in our patient, favor an autoinflammatory etiology.

Mortality from SCLS is often due to delayed diagnosis or misattribution to septic shock. Patients with severe EP should be monitored closely for signs of capillary leak. Early hallmarks include markedly elevated inflammatory biomarkers, edema, and worsening hypoalbuminemia that is out of proportion to nutritional status. Prompt recognition is critical for aggressive supportive care, such as hemodynamic support, volume management, and relief of compartment pressures by procedures like thoracentesis. Disease-specific therapy aimed at reducing cytokine production and subsequently reversing capillary leak is recommended. Glucocorticoids are effective in most cases of SCLS (3, 4). However, early use of glucocorticoids is limited, especially when a clear differential diagnosis from sepsis has not been established. In addition, high-dose intravenous immunoglobulin at 1 g/kg daily for two consecutive days represents the most promising therapeutic approach for both acute management and long-term prevention of SCLS (3). A recent case reported that SCLS associated with generalized pustular psoriasis was rapidly controlled by two 900-mg intravenous doses of spesolimab, an interleukin-36 receptor antagonist, administered 5 days apart (7).

In the aftermath of the SCLS episode, we switched to apremilast, a phosphodiesterase 4 inhibitor. The most notable clinical finding of this case is the dramatic and rapid therapeutic response with apremilast, which is faster than the efficacy reported in existing literature (29–32) and shows excellent safety in the treatment of severe EP complicated by SCLS. Apremilast acts by increasing intracellular cyclic adenosine monophosphate (cAMP) levels in immune cells, thereby reducing the release of proinflammatory cytokines and upregulating anti-inflammatory mediators (33). In addition, apremilast can alleviate vascular endothelial inflammation (34). Notably, apremilast has been confirmed to effectively control the cytokine storm in EP patients with COVID-19 pulmonary infection, and its safety and efficacy in the treatment of severe inflammatory diseases have been fully verified (35). Given that the loading dose of secukinumab exerts a prolonged therapeutic effect for at least 7 days, the drug remained within its therapeutic window when apremilast was initiated in this patient. Synergism of the two agents may contribute to the patient’s rapid clinical response.

Unlike cyclosporine and methotrexate, apremilast does not cause significant immunosuppression or end-organ toxicity. It has no contraindications for patients with active infections, active or previous malignancy, or severe hepatic dysfunction (36). This made it an attractive option for our patient, who was recovering from a critical illness with hepatic dysfunction. Our experience and the literature suggest that apremilast can induce remission even in severe cases of EP. It should be considered as a first-line alternative when traditional systemic agents or biologics are contraindicated.

## 4 Conclusion

In summary, this report adds a rare case of SCLS complicating EP to the literature. Fulminant EP is a dermatologic emergency associated with systemic cytokine-mediated conditions such as SCLS, which reflects underlying systemic immune dysregulation or hypersensitivity reactions rather than a single drug effect. Early hallmarks include markedly elevated inflammatory biomarkers, edema, and worsening hypoalbuminemia. These findings warrant clinical attention. Clinical management strategies should include fluid management combined with anti-inflammatory therapies, and close monitoring of patients receiving biologic agents, particularly those with severe inflammation or immune compromise. Sustained clinical vigilance and a personalized, cautious therapeutic approach are critical for patients with severe psoriasis, particularly those with acute fulminant EP. Ongoing research and case reporting will be invaluable to guide the safe use of biologics in EP.

## Data Availability

The original contributions presented in the study are included in the article/Supplementary Material. Further inquiries can be directed to the corresponding author.
